# Structural insights into simocyclinone as an antibiotic, effector ligand and substrate

**DOI:** 10.1093/femsre/fux055

**Published:** 2017-11-08

**Authors:** Mark J Buttner, Martin Schäfer, David M Lawson, Anthony Maxwell

**Affiliations:** 1 Department of Molecular Microbiology, John Innes Centre, Norwich Research Park, Norwich NR4 7UH, UK; 2 Department of Biochemistry, Duke University School of Medicine, Durham, NC 27710, USA; 3 Department of Biological Chemistry, John Innes Centre, Norwich Research Park, Norwich NR4 7UH, UK

**Keywords:** antibiotics, *Streptomyces*, DNA gyrase, aminocoumarins, DNA topoisomerases, transcription factor

## Abstract

Simocyclinones are antibiotics produced by *Streptomyces* and *Kitasatospora* species that inhibit the validated drug target DNA gyrase in a unique way, and they are thus of therapeutic interest. Structural approaches have revealed their mode of action, the inducible-efflux mechanism in the producing organism, and given insight into one step in their biosynthesis. The crystal structures of simocyclinones bound to their target (gyrase), the transcriptional repressor SimR and the biosynthetic enzyme SimC7 reveal fascinating insight into how molecular recognition is achieved with these three unrelated proteins.

## Teaser

Simocyclinones are actinomycete natural products that target bacterial DNA gyrase; structural work has revealed their molecular mode of action, aspects of their biosynthesis and the mechanism underlying their inducible export.

## INTRODUCTION

**Figure 1.**
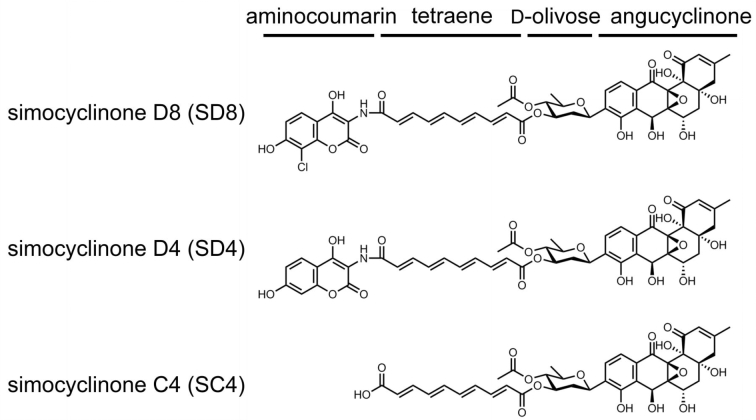
Chemical structures of simocyclinones D8, D4 and C4.

The increase in resistance to antimicrobials has become a serious challenge in the 21st Century, with rising antibiotic-resistant pathogens, particularly in hospital settings, and a paucity of new agents becoming available (Boucher *et al*. 2009; Bush *et al*. 2011). It is therefore essential that we continue our search for new antibacterial compounds, particularly novel natural products, which have the possibility of exploiting new chemical space. Actinomycetes, most notably the genus *Streptomyces*, have proved to be a rich source of bio-active molecules, with most antibiotics in current clinical use being actinomycete natural products or their derivatives (Clardy, Fischbach and Walsh 2006). In this review, we discuss the simocyclinones, natural products that were first isolated nearly 20 years ago from *Streptomyces antibioticus* Tü 6040, which produces simocyclinones A1, B1, B2, C2, C4, D4, D6, D7 and D8 (Schimana *et al*. 2000, 2001). More recently, *Kitasatospora* sp. and *Streptomyces* sp. NRRL B-24484 have been identified as producers of the novel simocyclinones D9, D10 and D11 (Bilyk *et al*. 2016); representative simocyclinones are shown in Fig. 1. As most work has been carried out on simocyclinone D8 (SD8), it will be the main topic of this review.

**Figure 2.**
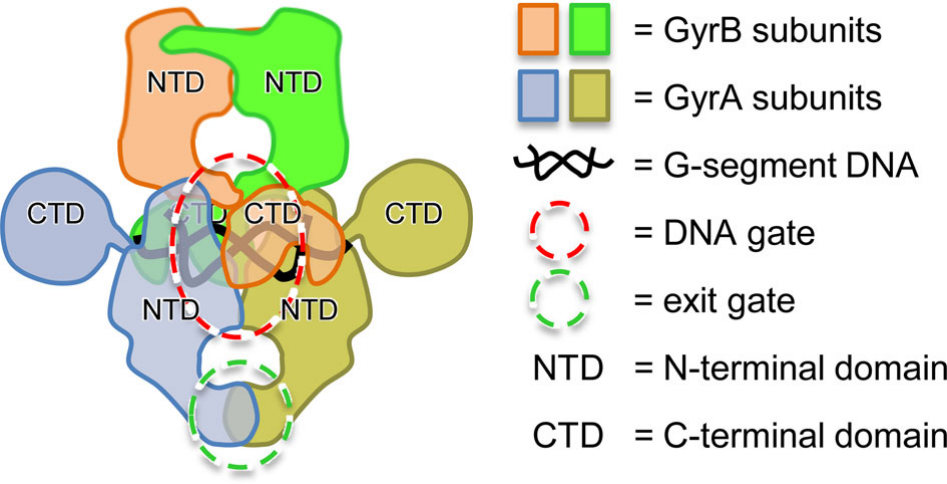
Schematic representation of the DNA gyrase A_2_B_2_ complex with bound G-segment DNA. Each subunit consists of an N-terminal domain (NTD) and a C-terminal domain (CTD). For reference, the DNA gyrase structure shown in Fig. 3 corresponds to a homodimer of a 55 kDa fragment of the GyrA NTD.

The target of simocyclinones is the type II DNA topoisomerase, DNA gyrase (Fig. 2). DNA topoisomerases (topos) are enzymes found in all organisms that catalyze the interconversions of different topological forms of DNA, e.g. relaxed–supercoiled, knotted–unknotted, catenated–decatenated (Vos *et al*. 2011; Bush, Evans-Roberts and Maxwell 2015) and are essential for DNA replication and transcription (Wang 2002). They are classified as type I or II depending upon whether their reactions proceed via single- or double-stranded breaks in DNA, and further divided into sub-types: IA, B, C and IIA and B, depending of mechanistic and evolutionary considerations (Wang 1996; Forterre *et al*. 2007). DNA gyrase (the target of simocyclinones) is a type IIA topoisomerase, and the only enzyme that can catalyze the introduction of negative supercoils into DNA. It is essential in all bacteria but lacking from animals, including humans, making it an ideal target for antibiotics. The type II topoisomerase in humans, topo II, has been developed as an anti-cancer target (Pommier *et al*. 2010) and can relax and decatenate DNA but cannot supercoil. Most bacteria, in addition to gyrase, have a second type II enzyme, topo IV, which is also a relaxing/decatenating enzyme, and is also a target for antibiotics.

DNA gyrase consists of two subunits, GyrA and GyrB, which form an A_2_B_2_ complex in the active enzyme (Fig. 2; Collin, Karkare and Maxwell 2011; Bush, Evans-Roberts and Maxwell 2015). The supercoiling reaction involves the wrapping of DNA around the A_2_B_2_ complex and the passage of one segment of DNA, termed the ‘T’ or ‘transported’ segment, through a double-stranded break in another, the ‘G’ or ‘gate’ segment. Catalytic supercoiling requires the hydrolysis of ATP. As this reaction proceeds via transient double-strand breaks in DNA, agents that can stabilize the broken DNA intermediate, such as the fluoroquinolones, are very effective antibacterial agents. A number of other compounds inhibit gyrase (and other topoisomerases) via this ‘cleavage-complex stabilization’ mechanism (Collin, Karkare and Maxwell 2011; Bush, Evans-Roberts and Maxwell 2015). In addition, gyrase and other type II topoisomerases can be inhibited by compounds that act at the ATP-binding site (Maxwell and Lawson 2003); this includes aminocoumarin antibiotics, such as novobiocin. As will be shown below, the inhibition of gyrase by simocyclinones occurs by a different, previously unknown, mechanism: they prevent the enzyme from binding DNA. It is possible that this mode of action can be exploited towards the development of novel, clinically relevant antibiotics. It is interesting to note that there are a number of peptide and protein inhibitors that exhibit the three mechanisms of gyrase inhibition (Collin, Karkare and Maxwell 2011). For example, microcin B17, CcdB and ParE can stabilize the cleavage complex, MfpA and Qnr proteins seem to prevent DNA binding, and FicT proteins modify GyrB and prevent ATPase activity (Harms *et al*. 2015).

Simocyclinones (D4 and D8) were discovered during the search for novel secondary metabolites from *Streptomyces* strains derived from soil samples (Schimana *et al*. 2000). These compounds showed antibiotic activity against certain Gram-positive bacteria and cytotoxic effects on tumor cell lines. By varying microbial growth and fermentation conditions the yield of these compounds was analyzed and optimized (Theobald, Schimana and Fiedler 2000; Schimana *et al*. 2001). Using 2D NMR, the structures of SD4 and SD8 were determined (Holzenkampfer *et al*. 2002) and shown to consist of an aminocoumarin moiety linked via a tetraene linker and olivose sugar to an angucyclinone polyketide moiety (Fig. 1). The presence of an aminocoumarin group and the discovery that some of the biosynthetic genes were related to those of the ‘classical’ aminocoumarin antibiotic novobiocin (Galm *et al*. 2002; Trefzer *et al*. 2002; see below), suggested that these compounds were likely to target bacterial DNA gyrase and that this was the likely cause of their antibacterial activity.

Simocyclinones have been studied most intensively as gyrase-inhibiting antibiotics, but the second section of this review covers the role of SD8 as an effector molecule controlling the activity of a transcription factor called SimR, responsible for linking the biosynthesis and export of SD8 in the producing organism, *S. antibioticus*. In addition, the SD8 precursor, 7-oxo-SD8, has been thoroughly characterized as a substrate for the enzyme SimC7, which reduces a carbonyl to a hydroxyl group at the C-7 position in the angucyclinone moiety of the molecule. This enzymatic step, which is critical because it converts an almost inactive precursor into the mature antibiotic, is covered in the third and final section of this review.

## SIMOCYCLINONES AS ANTIBIOTICS

### Activity of simocyclinones against bacteria

In general, the antibiotic activity of simocyclinones was found to be relatively weak, except against some Gram-positive bacterial species, for example *Bacillus brevis* (MIC 10 μg/ml) and *Streptomyces viridochromogenes* (MIC 1 μg/ml) (Schimana *et al*. 2000). Little activity was detected against Gram-negative bacteria. This is almost certainly due to the inability of simocyclinones to penetrate the outer membrane, since *imp* mutants of *E. coli*, which are specifically compromised in outer membrane integrity, become sensitive to SD8 (Edwards *et al*. 2009b), although multidrug efflux pumps like AcrB may also contribute to resistance (Oppegard *et al*. 2009). However, it has been pointed out that most of these susceptibility tests have been carried out using stock lab strains, and SD8 has shown more promising activity against some clinical isolates of *E. coli* and *Klebsiella pneumoniae* (Richter *et al*. 2010). More recently, the discovery of new simocyclinones (Bilyk *et al*. 2016) and the capacity for engineering novel compounds, as has been carried out with the classical aminocoumarins (Heide *et al*. 2008; Heide 2009, 2014) and to a limited extent with simocyclinones (Anderle *et al*. 2007a,b), has raised the possibility of compounds with increased antibacterial potency. However, given that SD8 has been shown to inhibit human topo II (Flatman *et al*. 2005; Sadiq *et al*. 2009), the potential for mammalian toxicity must be borne in mind.

### How simocyclinones inhibit DNA gyrase

The similarity between the structures of simocyclinones (Fig. 1) and those of the classical aminocoumarins led to the expectation that simocyclinones would inhibit bacterial DNA gyrase by competitively binding to the ATPase active site in the GyrB subunit. It was shown that simocyclinone D8 (and D4) did indeed inhibit DNA supercoiling catalyzed by *E. coli* gyrase but, surprisingly, also inhibited DNA relaxation (Flatman *et al*. 2005), an ATP-independent reaction. Moreover, ATPase assays showed that SD8 and SD4 did not inhibit this reaction under conditions where novobiocin was effective. The most common mode of action of topoisomerase-targeted drugs (e.g. fluoroquinolones) is the stabilization of the enzyme-DNA cleavage complex. It was shown that SD8 did not act in this way but was found to antagonize the ability of fluoroquinolones, and other agents, to induce cleavage-complex formation (Flatman *et al*. 2005).

Taken together, these data suggested that simocyclinones might interfere with the binding of gyrase to DNA rather than to ATP. This was directly tested using surface-plasmon resonance (SPR) in which DNA was tethered to the chip surface and the binding of gyrase monitored in the absence and presence of SD8 (Flatman *et al*. 2005). The presence of SD8 at relatively low concentrations (50 nM) blocked DNA binding. When different domains of gyrase were examined for their ability to bind SD8 using SPR and isothermal titration calorimetry (ITC), it was found that interaction occurred only with the N-terminal domain of GyrA (Flatman *et al*. 2005), which was already known to contain the binding site for the G-segment DNA (Morais Cabral *et al*. 1997). These biochemical and biophysical experiments therefore supported the idea that simocyclinones act by binding to the GyrA subunit at a DNA-binding site to prevent the binding of the enzyme to DNA; a completely novel mode of action. This idea was later corroborated by X-ray crystallography (Edwards *et al*. 2009b, Hearnshaw *et al*. 2014), see below. Binding of SD8 to the N-terminal domain of GyrA was also seen using circular dichroism experiments (Sissi *et al*. 2010); this method also showed evidence for a second binding site in the C-terminal domain of GyrB, albeit of lower affinity than the GyrA-binding site. Subsequent ITC experiments (Hearnshaw *et al*. 2014) also found evidence for a binding site in GyrB, but estimated that it was ∼1000-fold weaker than the GyrA site; it is unlikely that the GyrB site contributes to the activity of simocyclinones.

SD8 has also been found to inhibit *E. coli* topo IV and human topo II, albeit with a lower potency than against gyrase (Flatman *et al*. 2005; Sadiq *et al*. 2009). In other work, SD8 was found to also inhibit *S. aureus* gyrase, but was much less effective against topo IV from *E. coli* and *S. aureus* (Oppegard *et al*. 2009); *S. aureus* gyrase was found to be 3–4-fold less sensitive to SD8 than *E. coli* gyrase. Elsewhere, it was found that the difference in SD8 potencies between these enzymes was ∼20-fold (Alt *et al*. 2011); however, it should be stressed that the absolute IC_50_ values are likely to be affected by assay conditions, which differ between the two enzymes. Taken together, it seems that gyrase is the preferred target for simocyclinones, and that they act by binding to the GyrA subunit of gyrase preventing the binding of DNA.

### How simocylinone D8 binds to gyrase

Biochemical and biophysical data (described above) strongly suggested that the simocyclinones bind to GyrA in a region of the protein involved in DNA binding. This proposal was confirmed by X-ray crystallography. Crystallization trials using simocyclinone D8 (SD8) and the N-terminal domain of the DNA gyrase A protein (GyrA59), whose structure was already known (Morais Cabral *et al*. 1997), gave diffracting crystals (Edwards *et al*. 2009a). This first structure (initially solved at 2.6-Å resolution) revealed a tetramer of GyrA59 that consisted of two GyrA59 dimers cross-linked by four molecules of SD8 (Edwards *et al*. 2009b). Two binding pockets were observed for SD8 in each subunit, both lying within the DNA-binding ‘saddle’ (Morais Cabral *et al*. 1997) of the GyrA59 dimer, one accommodating the aminocoumarin moiety and the other accommodating the angucyclinone moiety. Selection of spontaneous SD8-resistant *E. coli* mutants showed that the mutations occurred in both pockets, corroborating the crystal structure (Edwards *et al*. 2009b). Further site-directed mutants also supported the structure, while others could not be fully rationalized (see below), suggesting that this structure might not reflect the situation *in vivo*. Although the crystal structure showed a protein tetramer, it was suspected that this dimer–dimer interaction was stabilized in the crystal and may not represent the physiologically relevant form of the complex.

**Figure 3.**
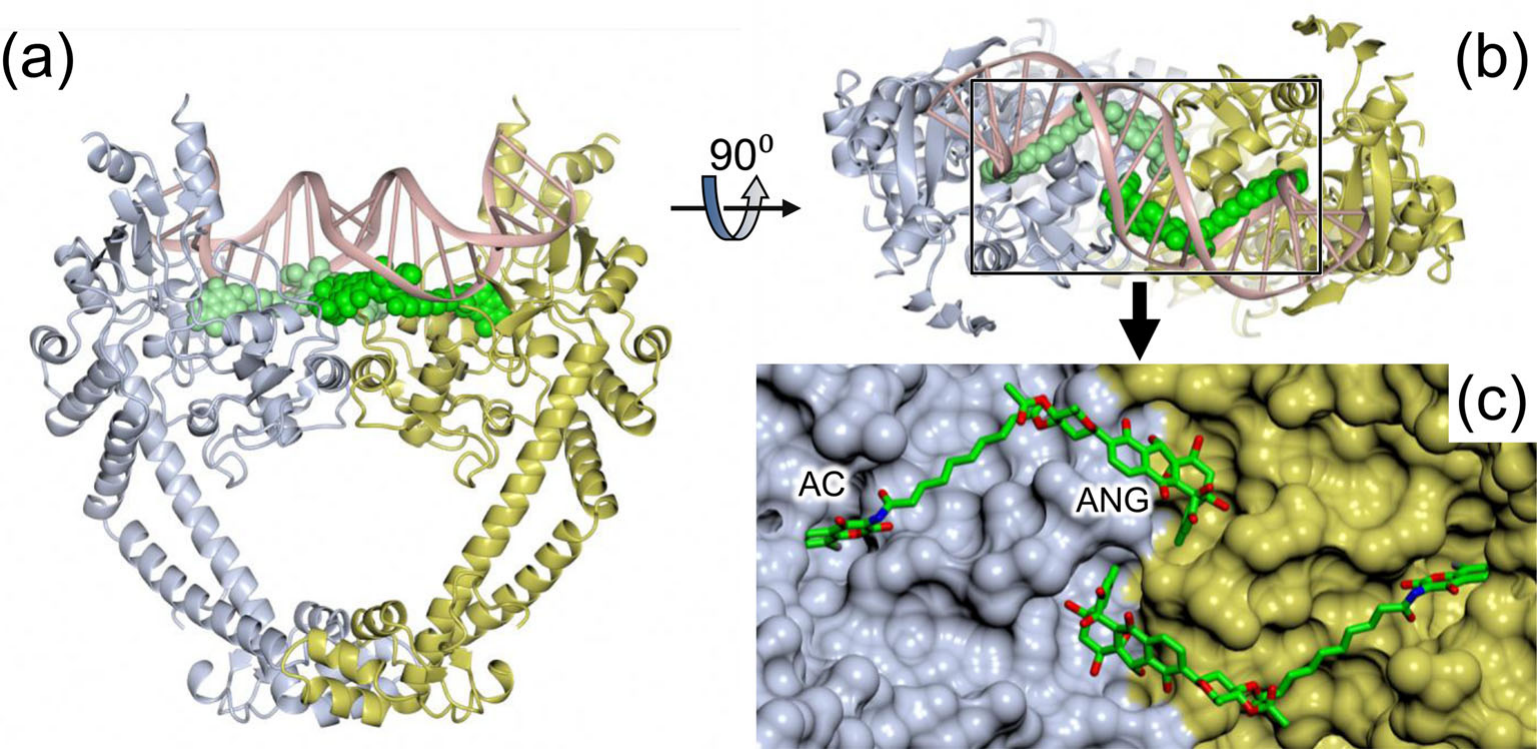
(**a**) Structure of *E. coli* DNA GyrA homodimer (55 kDa N-terminal fragment) with two molecules of SD8 bound; one subunit is colored blue and the other in yellow; the SD8 molecules are shown in two shades of green (PDB accession number 4CKL). A DNA duplex taken from a superposed structure of a *Staphylococcus aureus* gyrase-DNA-drug complex (PDB accession number: 2XCS) is also shown in pink to illustrate that SD8 would block the interaction of G-segment DNA with the DNA-binding ‘saddle’. (**b**) Top view of panel a, looking down the dimer 2-fold axis. (**c**) Enlarged view of the boxed region shown in panel b, with the SD8 ligands in stick representation with atom coloration (carbon, green; oxygen, red; nitrogen, blue, chlorine, gray). This clearly shows that the antibiotic spans the dimer interface with distinct binding pockets for the terminal angucyclinone (ANG) and aminocoumarin (AC) groups. (This figure and the other structural figures were prepared using CC4MG; McNicholas *et al*. 2011.)

**Figure 4.**
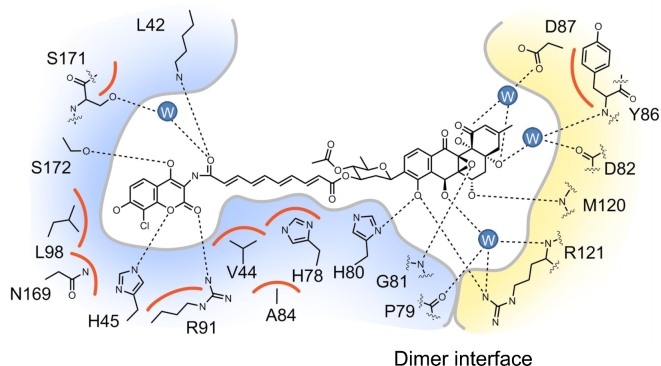
Schematic representation of the SD8-binding pocket of GyrA showing all residues within 4 Å of the ligand, as revealed in the crystal structure of the GyrA-SD8 complex (PDB accession number 4CKL). One subunit is shown in blue and the other in yellow. Hydrogen bonds are indicated by dotted lines; van der Waal contacts are indicated by orange arcs, and water molecules are shown as filled blue circles labeled ‘W’. For clarity, all hydrogens have been omitted.

Analysis of the SD8-GyrA59 complex using nanoelectrospray ionization mass spectrometry showed that the tetrameric species observed in the crystal could be reproduced in solution, but only at high SD8 concentrations, while at lower concentrations, a dimeric species was present with two SD8 molecules bound per dimer; this result was potentially at odds with the previous structural data (Edwards *et al*. 2009b). Further mass spectrometry suggested that the binding of SD8 to the protein dimer showed strong allosteric cooperativity (Edwards *et al*. 2011). A subsequent crystal structure of a shorter version of the N-terminal domain of GyrA (GyrA55), which lacks residues that stabilize dimer–dimer interactions in the tetramer, revealed a discrete protein dimer with two SD8 molecules bound (Fig. 3; Hearnshaw *et al*. 2014). This structure, solved at 2.05-Å resolution, proved to be entirely consistent with all the mutations to SD8 resistance that had been previously made or selected (Edwards *et al*. 2009b); additional mutants made in response to the revised structure were also shown to be consistent (Hearnshaw *et al*. 2014). In addition to the new structure being dimeric, rather than tetrameric, the conformation of SD8 is significantly different, compared with the earlier structure: the orientation of the aminocoumarin within the aminocoumarin pocket is somewhat different, while the angucyclinone ‘pocket’ has shifted such that it now spans the interface between the two monomers (Fig. 4) and thus could provide a structural explanation for the cooperative binding observed by mass spectrometry. This new position for the angucyclinone group suggests that the binding of SD8 effectively ‘staples’ the GyrA dimer closed so inhibiting the conformational changes that need to occur upon DNA binding and cleavage (Hearnshaw *et al*. 2014).

The SD8-binding site on gyrase is close to the binding site of the fluoroquinolone antibiotics (Laponogov *et al*. 2009; Bax *et al*. 2010; Laponogov *et al*. 2010), raising the possibility of generating hybrid compounds. To this end, a series of ciprofloxacin-aminocoumarin hybrids has been synthesized, designed to bind to the aminocoumarin pocket of SD8 and to the fluoroquinolone pocket (Austin *et al*. 2016); some of these compounds retain good inhibitory activity against gyrase. It remains to be seen whether such compounds can be developed as viable antibiotics. Also flavone-base analogs of simocyclinones have been made in order to bind to a hydrophobic cleft in the protein and further stabilize binding (Verghese *et al*. 2013). Although some of these compounds are effective gyrase inhibitors, they also stabilize the gyrase-DNA cleavage complex and probably act via a mechanism involving DNA intercalation, i.e. they do not bind at the intended site.

Taken together, we conclude that simocyclinones bind to the A subunit of DNA gyrase, in a region that is normally occupied by the G-segment DNA (Figs 2–4) and prevent the initial interaction of DNA with the enzyme and thus all the ensuing catalytic events. This is quite distinct from the mode of action of fluoroquinolones (cleavage-complex stabilization) and aminocoumarins (competitive inhibitors of ATP binding) and raises the possibility of developing other agents that use this mode of action, which would be less likely to be cross-resistant to known antibiotics.

## SIMOCYCLINONE D8 AS A TRANSCRIPTION FACTOR EFFECTOR MOLECULE

SD8 has been studied most intensively as a gyrase-inhibiting antibiotic. However, it has also been characterized as an effector molecule controlling the activity of a transcription factor called SimR, responsible for linking the biosynthesis and export of SD8 in the producing organism, *S. antibioticus* (Le *et al*. 2009, 2011a,b). It is perhaps under-appreciated that antibiotics are often potentially toxic to the organisms that produce them (Cundliffe 1989; Hopwood 2007). Therefore, producing organisms must have mechanisms to ensure that the antibiotic export machinery is in place when antibiotic biosynthesis begins. The relevant mechanism in the simocyclinone producer is specified by two adjacent genes, *simR* and *simX*, which sit within the simocyclinone (*sim*) biosynthetic gene cluster (Galm *et al*. 2002; Trefzer *et al*. 2002; Le *et al*. 2009). The SimR and SimX proteins resemble the TetR/TetA repressor/efflux pump pair found in a number of human pathogens, which confer resistance to clinically important tetracyclines (Chopra and Roberts 2001). SimX is an efflux pump, a member of the major facilitator superfamily, which exports simocyclinone from the producing organism. *simX* transcription is repressed by SimR, a TetR-family transcriptional regulator (TFR) that binds to two separate operators in the intergenic region between the divergently transcribed *simR* and *simX* genes (Le *et al*. 2009). Simocyclinone abolishes DNA-binding by SimR, thereby derepressing transcription of the *simX* efflux pump gene, and this provides the mechanism that couples the biosynthesis of simocyclinone to its export. It was also shown that the biosynthetic intermediate simocyclinone C4 (SC4; Fig. 1), could dissociate SimR from its operators (Le *et al*. 2009). Subsequently, crystal structures of SimR alone (apo; 1.95-Å resolution) (Le *et al*. 2011b), in complex with its operator DNA (2.99-Å resolution) (Le *et al*. 2011a), and in complex with either SD8 or SC4 (both 2.3-Å resolution) (Le *et al*. 2011b), showed how SimR binds its effector ligand and how ligand binding prevents SimR from binding to its operator DNA. Unsurprisingly, there is no similarity between the ligand-binding pockets in gyrase and SimR.

### How SimR binds SD8

**Figure 5.**
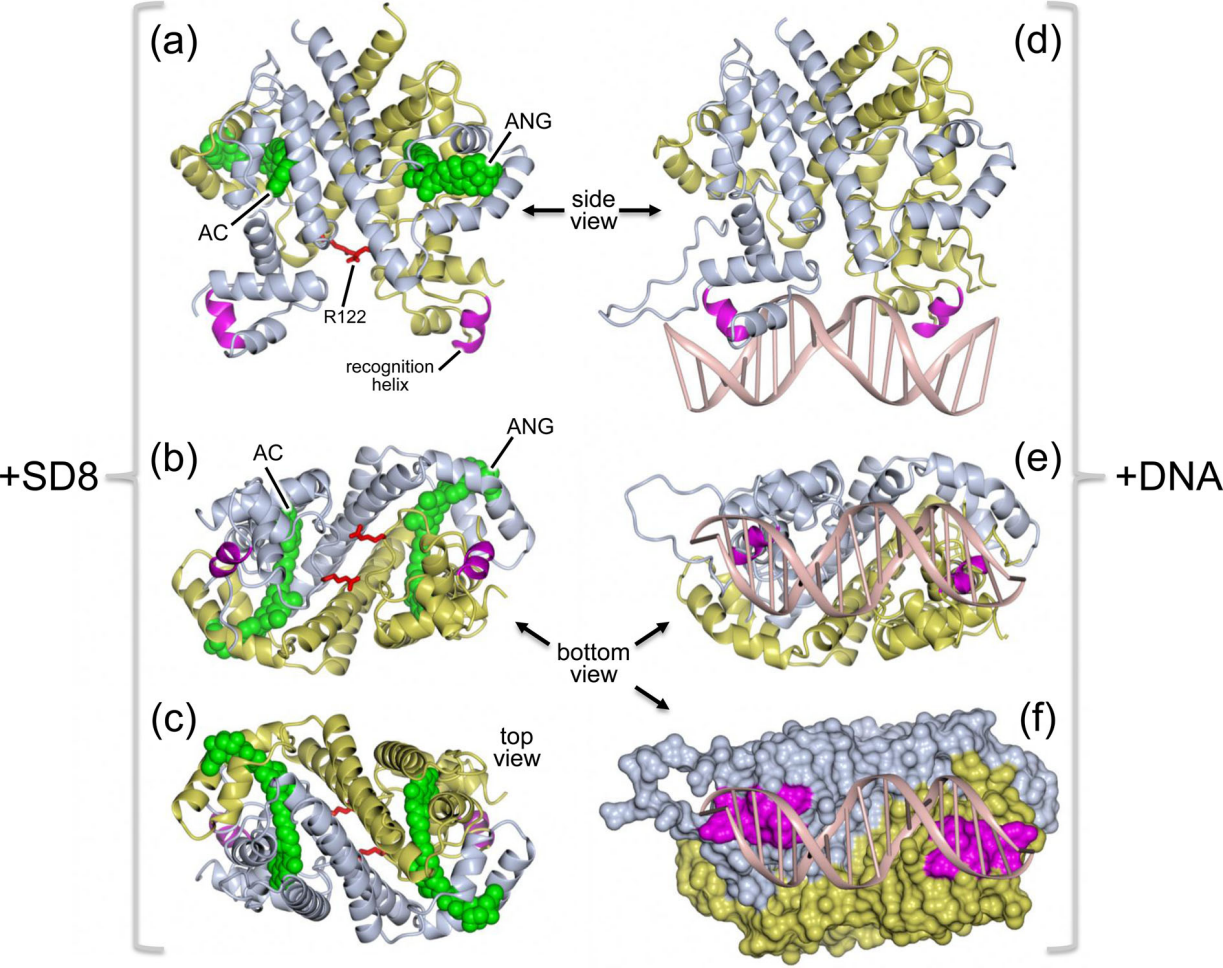
Comparison of the SimR-SD8 (**a,b,c**) and SimR-DNA (**d,e,f**) structures with one SimR subunit shown in blue and the other shown in yellow. The two recognition helices are highlighted in magenta and bound SD8 molecules are shown in green. Note that the ligand-binding pocket in SimR spans the two protein subunits, with the angucyclinone (ANG) end of SD8 bound in one subunit while the aminocoumarin (AC) end is bound in the other such that SD8 skewers the two subunits. Note also that in the apo form of SimR (structure not shown), Arg122 is buried in its cognate subunit; however, in the SimR-SD8 complex, each copy of this residue (shown as red sticks) projects across the dimer interface into a pocket in the surface of the opposing subunit. Arg122 is not ordered in the SimR-DNA structure. (PDB accession numbers: SimR-SD8: 2Y30; SimR-DNA: 3ZQL; SimR-apo: 2Y2Z).

**Figure 6.**
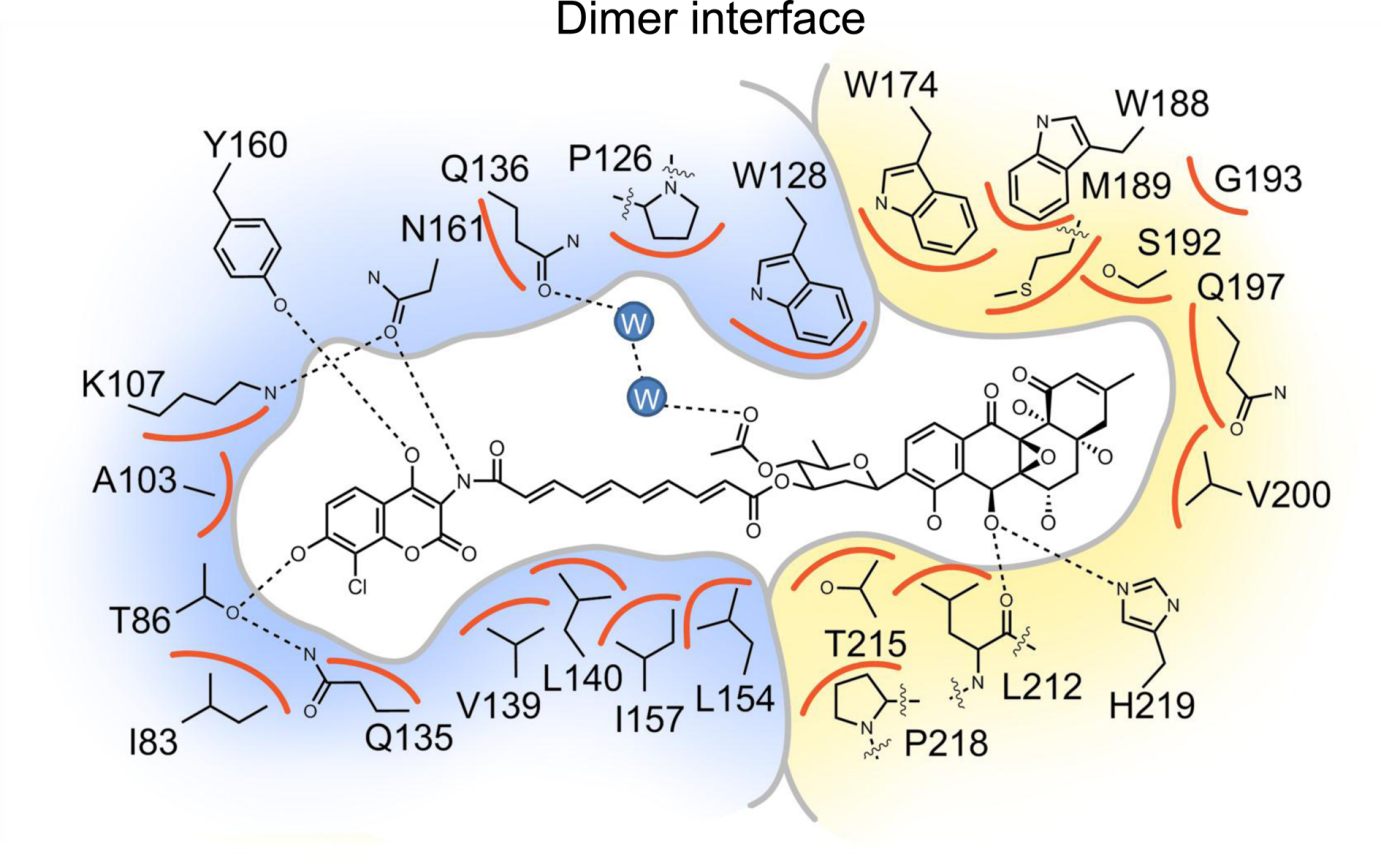
Schematic representation of the SD8-binding pocket of SimR showing all residues within 4 Å of the ligand. One subunit is shown in blue and the other shown in yellow. Hydrogen bonds are indicated by dotted lines and van der Waal contacts are indicated by orange arcs. The two water molecules that link Gln136 to the olivose sugar are shown as filled blue circles labeled ‘W’. For clarity, all hydrogens have been omitted.

TFRs function as homodimers, with each subunit having two domains, an N-terminal DNA-binding domain (DBD) containing a helix-turn-helix (HTH) motif, and a C-terminal ligand-binding domain (LBD) (Ramos *et al*. 2005; Yu *et al*. 2010; Cuthbertson and Nodwell 2013). The ligand-binding pocket of SimR is unusual; in other characterized TFRs, one ligand-binding pocket is typically contained within each subunit and so, for example, in the closely related protein, ActR, there is only one ligand contact with the second subunit (Willems *et al*. 2008), while in TetR itself there is none (Orth *et al*. 2000). In contrast, the ligand-binding pocket in SimR spans the two protein subunits, with the angucyclinone of SD8 bound in one subunit, while the olivose sugar, tetraene and aminocoumarin parts of the molecule are bound in the other (Le *et al*. 2011b) (Figs 5 and 6). This split binding pocket is ∼30 Å in length, with SD8 bound in an extended conformation. Although SD8 has 19 atoms that could potentially participate in hydrogen bonding, there are only five direct hydrogen bonds between SimR and SD8, three with the aminocoumarin and two with the angucyclinone (Fig. 6). However, the dearth of hydrogen bonding is compensated for by extensive van der Waals contacts with the protein along the length of the ligand (Fig. 6). The way cognate ligands are bound by TFRs is highly variable. For example, when the SimR-SD8 structure is compared with that of the complex between the closely related TFR protein ActR and its cognate ligand, the antibiotic actinorhodin, the long axis of the actinorhodin molecule lies almost perpendicular to that of SD8 in the SimR–SD8 structure (Willems *et al*. 2008; Le *et al*. 2011b).

### How simocyclinone D8 prevents SimR from binding DNA

Available evidence suggests that apo-TFRs sample a range of conformations in solution and that ligand binding simply captures one of these conformations, rather than inducing the conformational change (Reichheld, Yu and Davidson 2009; Yu *et al*. 2010; Cuthbertson and Nodwell 2013). SimR-apo did not crystallize in its DNA-binding form (apparent from the distance between its recognition helices), and indeed this is generally true of TFR apo-proteins (Yu *et al*. 2010). However, comparison of the SimR-apo, SimR-SD8 and SimR-DNA structures provided clear insight into the likely mechanism of ligand-mediated derepression.

**Figure 7.**
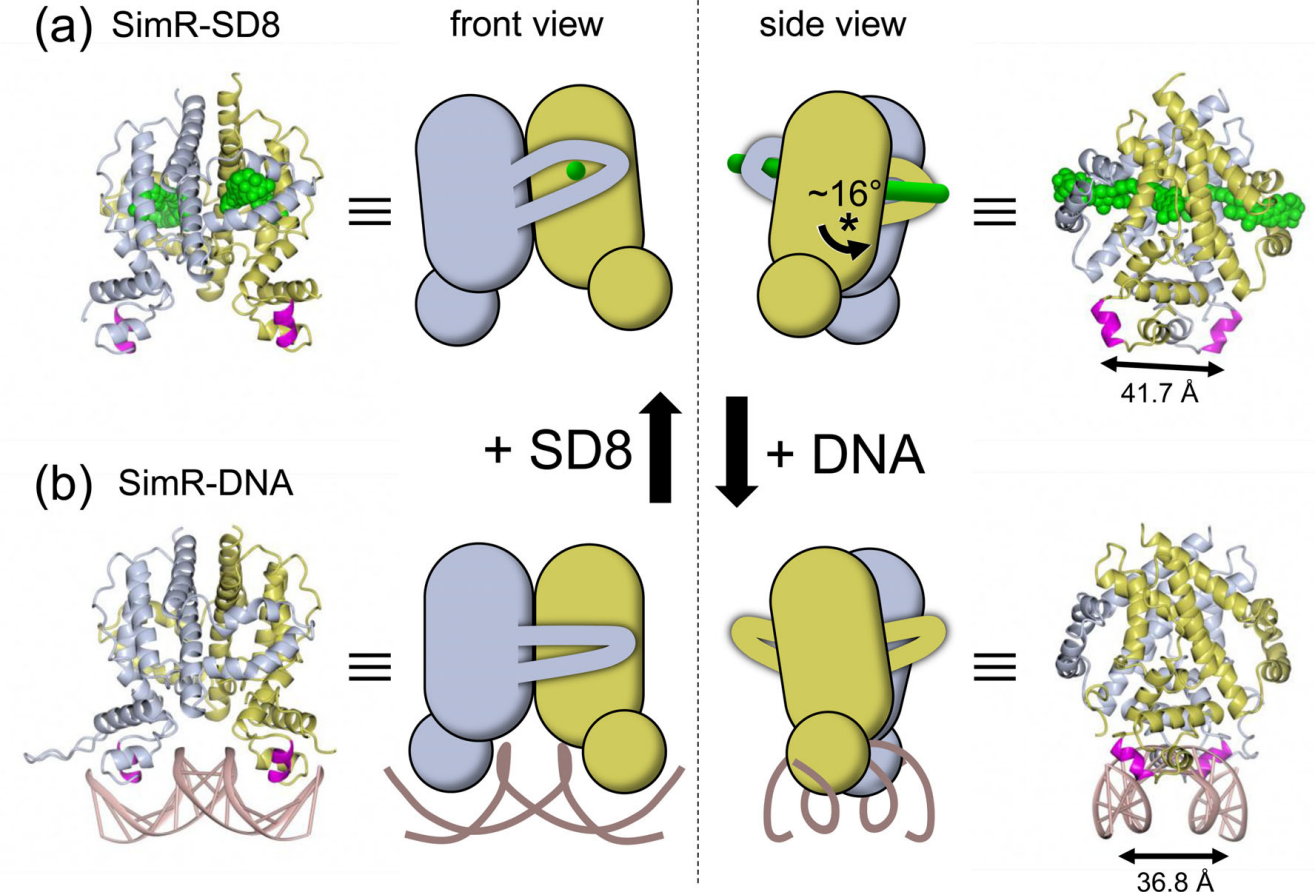
Structures of (**a**) SimR-SD8 and (**b**) SimR-DNA together with schematic representations illustrating the rigid-body rotation of the subunits relative to one another. To emphasize the subunit rotation, the position of the blue subunit is fixed in each panel so that the rotation of the yellow subunit accounts for all the movement in the dimer. The asterisk indicates the pivot point around which rotation occurs. Note that the net effect of subunit rotation is that the distance separating the two recognition helices increases to 41.7 Å in the SD8-bound form, a distance incompatible with DNA binding. Note also that helices α9–α10 form a wrapping arm that engages the LBD of the opposing subunit and that these helices additionally form the angucyclinone end of the ligand-binding pocket. (PDB accession numbers: SimR-SD8: 2Y30; SimR-DNA: 3ZQL).

The ligand-binding sites of TFRs are remote from their DBDs and derepression generally involves allosteric mechanisms (Ramos *et al*. 2005; Yu *et al*. 2010; Cuthbertson and Nodwell 2013). Ligand-bound and DNA-bound structures have been determined for several TFRs, including QacR, DesT, CgmR and TetR itself, and in these cases conformational changes appear to be transmitted largely within the same subunit (Orth, Saenger and Hinrichs 1999; Orth *et al*. 2000; Schumacher *et al*. 2001; 2002; Itou *et al*. 2010; Miller *et al*. 2010). Specifically, they suggest that ligand binding traps a conformational state in which the DBD (in particular the HTH motif) is repositioned relative to the LBD such that the two recognition helices in the homodimer are too far apart to bind appropriately in consecutive major grooves of the DNA. In contrast, comparison of the repressive SimR-DNA structure with the derepressed SimR-SD8 structure shows that the relative dispositions of the LBDs and DBDs within each individual SimR subunits remain essentially unchanged on ligand binding. Instead, SD8 binding captures a conformation in which there is a rigid-body rotation of one SimR subunit relative to the other, and this rigid-body rotation moves the recognition helices ∼5 Å further apart in the derepressed (SD8-bound) state, preventing DNA binding (Fig. 7). It may well be significant that the ligand-binding sites in the previously characterized TFRs are contained almost entirely within individual subunits, whereas the ligand-binding pocket in SimR spans the two subunits.

Two helices of the SimR LBD (α9-α10) form a wrapping arm that folds around the LBD of the opposing subunit (Figs 5 and 7). These two helices form the end of the ligand-binding pocket responsible for binding the angucyclinone of SD8 (Figs 5, 6 and 7), and the wrapping arm changes conformation in the ligand-bound state. Only five reciprocal inter-subunit hydrogen bonds (i.e. 10 in total) are maintained between the repressive DNA-bound conformation and the derepressed ligand-bound structure, and all five of these link the wrapping arm with the LBD of the other subunit. As a consequence, when the subunits rotate in the ligand-bound form, the wrapping arm moves with them. Because the ligand-binding pocket passes through both subunits, SD8 effectively skewers the dimer, rigidifying the complex, and because it is a relatively hydrophobic molecule, SD8 contributes to the hydrophobic core of the SimR dimer, stabilizing the overall structure. In addition, in the apo and DNA-bound structures, the two SimR subunits present essentially flat surfaces to one another, allowing them to rotate relative to each other. In contrast, in the SD8-bound form, the side-chain of Arg122 from each subunit projects across the dimer interface into a pocket in the surface of the opposing subunit, potentially acting as locating pins to lock the subunits together (Fig. 5).

The biosynthetic intermediate simocylinone C4 (SC4) lacks the aminocoumarin ring present in the mature antibiotic (Fig. 1) and is essentially inactive as a DNA gyrase inhibitor; the SD8 IC_50_ is 0.1 μM, whereas the SC4 IC_50_ is >100 μM (Edwards *et al*. 2009b). However, despite the absence of the aminocoumarin ring, SC4 binds SimR and prevents it from binding DNA (Le *et al*. 2009). The structure of the SimR-SC4 complex has also been determined (Le *et al*. 2011b). Comparison of the SD8-SimR and SC4-SimR structures shows that the two molecules bind SimR in the same way, meaning the parts common to both molecules (the angucyclinone, tetraene and olivose sugar) occupy equivalent positions in the binding pocket. SC4 is slightly less effective than SD8 at derepressing SimR *in vitro* (Le *et al*. 2009) and this is probably a consequence of the fewer favorable interactions that SC4 makes with the protein, due to the absence of the aminocoumarin. These results show that a pathway intermediate that is not an active antibiotic can induce expression of the efflux pump, and similar observations have been made in other antibiotic pathways, particularly for actinorhodin (Otten, Ferguson and Hutchinson 1995; Jiang and Hutchinson 2006; Ahn *et al*. 2007; Tahlan *et al*. 2007; Ostash *et al*. 2008; Tahlan *et al*. 2008; Willems *et al*. 2008). These data raise the possibility of a ‘feed-forward’ mechanism, in which inactive intermediates ensure expression of the efflux pump prior to the build-up of a toxic concentration of the potentially lethal mature antibiotic (Hopwood 2007; Tahlan *et al*. 2007; Le *et al*. 2009).

## 7-OXO-SIMOCYCLINONE D8 AS A SUBSTRATE

While the functions of most of the biosynthetic enzymes encoded within the *S. antibioticus sim* cluster have been predicted (Galm *et al*. 2002; Trefzer *et al*. 2002), the biosynthetic pathway remains largely uncharacterized experimentally. This lack of knowledge about the biosynthesis of simocyclinones is well illustrated by the tetraene moiety. Trefzer *et al*. (2002) proposed that the tetraene linker would be the product of the large modular type I polyketide synthase (PKS), SimC1ABC, working *in trans* with two monofunctional enzymes, SimC6 and SimC7. Yet when Bilyk *et al*. (2016) sequenced the *Kitasatospora* sp. and *Streptomyces* sp. NRRL B-24484 biosynthetic clusters, there were no type I PKS genes present, and the tetraene was instead shown to be synthesized by an iterative type II PKS. This type II PKS is also present in *S. antibioticus*, leaving the role of the type I PKS unknown. To date, only two biosynthetic enzymes have been characterized biochemically: SimL and SimC7. SimL catalyses the presumed last step in the pathway, acting as an amide bond-forming ligase that attaches the aminocoumarin to the tetraene linker (Luft *et al*. 2005; Pacholec *et al*. 2005; Anderle *et al*. 2007b).

**Figure 8.**
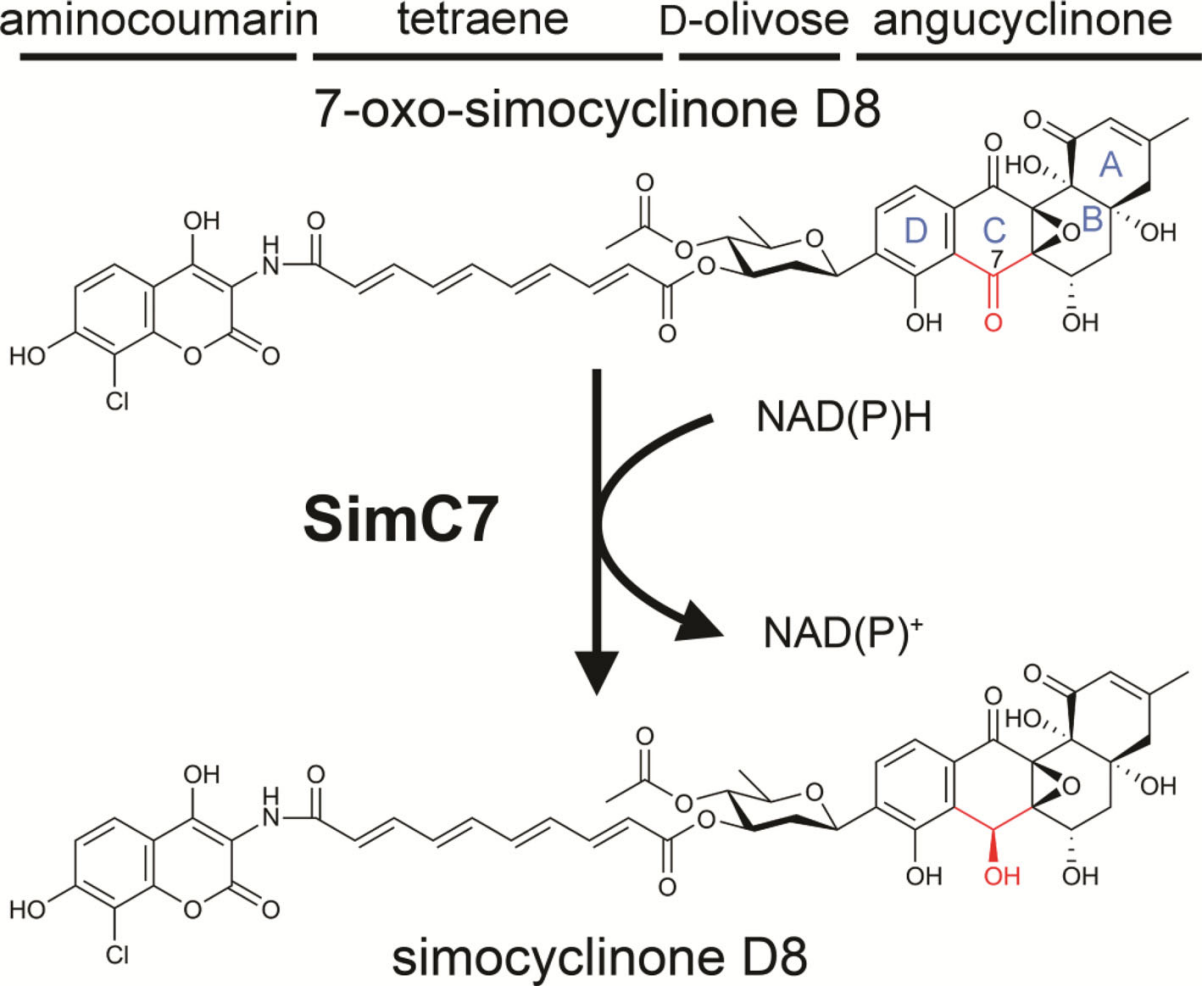
SimC7 catalyzes the reduction of 7-oxo SD8 to simocyclinone D8. Labels A-D denote the four rings of the angucyclinone; the C-7 carbonyl/hydroxyl is highlighted in red.

As noted above, the second enzyme, SimC7, was originally proposed to be involved in the biosynthesis of the tetraene linker. It was subsequently shown to be an NAD(P)H-dependent ketoreductase that catalyzes the reduction of a carbonyl to a hydroxyl group at the C-7 position of the angucyclinone, highlighting the dangers of relying on speculative gene annotations (Fig. 8; Schäfer *et al*. 2015). This enzymatic step is essential for antibiotic activity, converting the almost inactive 7-oxo-SD8 (IC_50_ ∼ 50–100 μM) into the potent gyrase inhibitor SD8 (IC_50_ ∼ 0.1–0.6 μM) (Schäfer *et al*. 2015). Based on the intermediates produced by *S. antibioticus*, it seems the biosynthesis of SD8 starts with assembly of the angucyclinone, followed by the attachment of the olivose sugar, and then the tetraene linker, and lastly the aminocoumarin (i.e. as drawn in Figs 1 and 8, SD8 is assembled from right to left) (Schimana *et al*. 2001). Therefore, the natural substrate of SimC7 is probably a 7-oxo angucyclinone intermediate lacking the attached olivose sugar, tetraene linker and aminocoumarin, an intermediate that is detectable only in *ΔsimC7* mutants (Schäfer *et al*. 2015). Nevertheless, the enzyme readily accepts as a substrate the full-length intermediate 7-oxo-SD8, the product made by *ΔsimC7* mutants (Schäfer *et al*. 2015).

SimC7 is a member of the short-chain dehydrogenase/reductase (SDR) superfamily. These proteins have diverse biochemical activities, including functioning as dehydratases, reductases, epimerases, dehydrogenases and decarboxylases (Kallberg, Oppermann and Persson 2010; Persson and Kallberg 2013). Classical SDR enzymes have a characteristic Ser-Tyr-Lys catalytic triad in their active site, in which the latter two residues form a YxxxK motif. The conserved tyrosine acts as a central acid-base catalyst that donates a proton to the substrate. The adjacent lysine serves to lower the pKa of the tyrosine hydroxyl group and often contributes directly to a proton relay mechanism. Lastly, the hydroxyl group of the serine polarizes the carbonyl group of the substrate (Kavanagh *et al*. 2008). The catalytic mechanism of SimC7 was investigated because it shares little sequence similarity with other characterized ketoreductases, even with functionally analogous polyketide ketoreductases involved in the biosynthesis of related angucyclinone antibiotics. Most of all, alignments of SimC7 with other SDR proteins suggested that SimC7 lacked the classical catalytic triad, including the tyrosine that acts as the central acid-base catalyst in classical SDR proteins. This possibility was investigated by determining the structures of SimC7 alone (apo; 1.6-Å resolution), the binary complex with NADP^+^ (1.95-Å resolution) and the ternary complex with both NADP^+^ and 7-oxo-SD8 (1.2-Å resolution) (Schäfer *et al*. 2016). As might be expected, there is no similarity between the simocyclinone-binding pockets in gyrase, SimR and SimC7.

**Figure 9.**
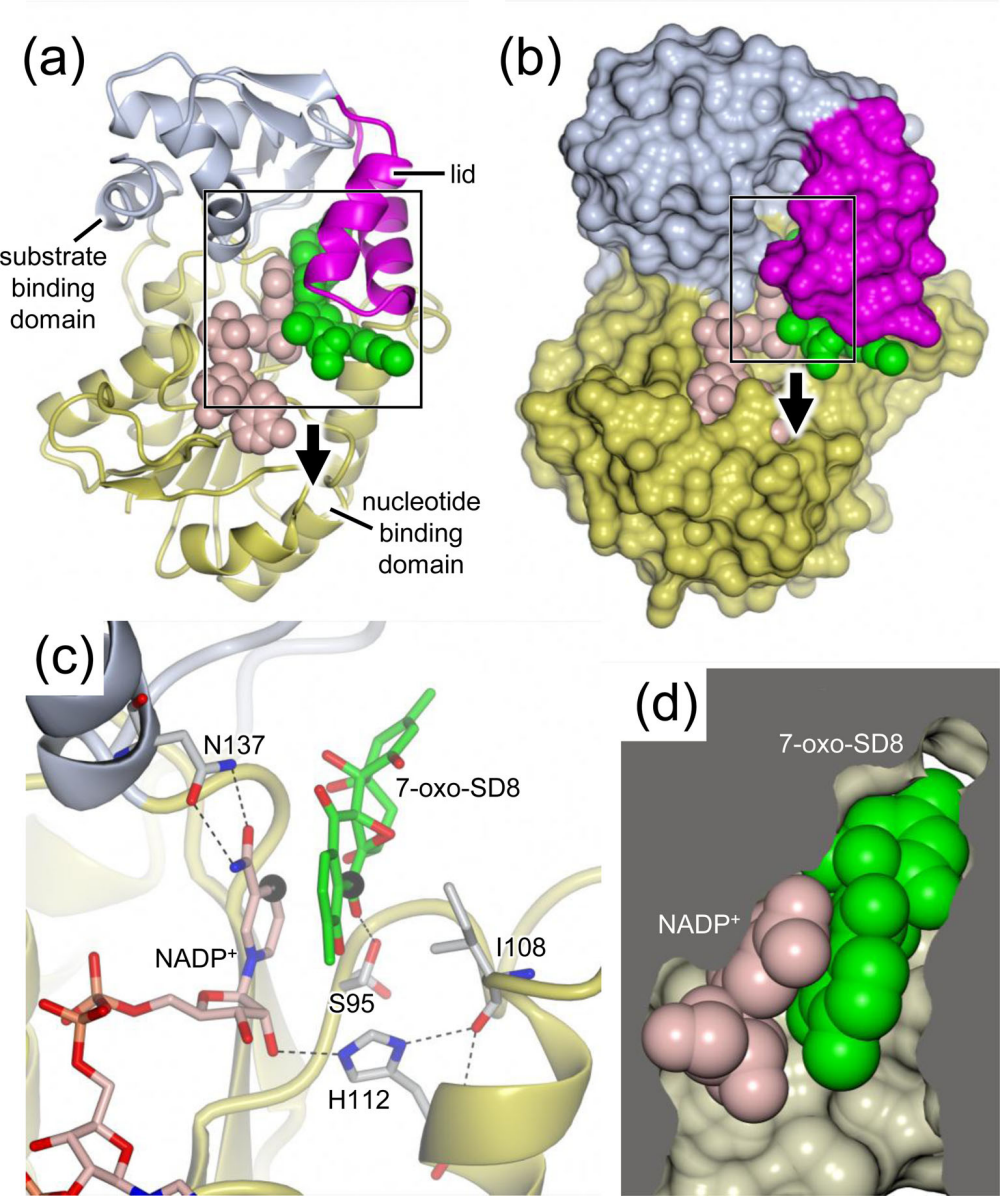
(**a**) and (**b**) Crystal structure of the SimC7 ternary complex with NADP^+^ and 7-oxo-SD8. The nucleotide-binding domain, the substrate-binding domain and the lid motif are shown in yellow, blue and magenta, respectively. NADP^+^ is shown in pink and 7-oxo-SD8 is shown in green. For the latter, only the crystallographically resolved atoms are shown, i.e. the angucyclinone, the olivose and roughly half of the tetraene linker. (**c**) Close-up showing the active site of the ternary complex including the Ser95-Ile108-His112 ‘catalytic triad’ residues, and Asn137, which is important in maintaining the *syn*-conformation of the cofactor. C-4 of the cofactor nicotinamide ring and C-7 of the substrate are highlighted by black spheres, which are 3 Å apart, indicating that the substrate is exactly positioned for direct hydride transfer. (**d**) Cross-section through the active site pocket, showing how tightly the cofactor (pink) and substrate (green) are bound. For clarity, only the nicotinamide ribosyl moiety of the cofactor is shown in panel d, and only the angucyclinone moiety of the substrate is shown in panels c and d (PDB accession number: 5L4L).

SimC7 has two domains (Fig. 9), a larger Rossmann-fold domain that binds NADP^+^ and a smaller substrate-binding domain that is characteristic of the so-called extended SDR subfamily (Kavanagh *et al*. 2008). This latter domain contains a ‘lid’ motif consisting of two anti-parallel α-helices that sits over the active site. The apo, binary and ternary SimC7 structures are very similar except for the orientation of this lid, which closes somewhat over the bound substrate (maximum Cα-Cα shift 5.35 Å). The underside of the lid forms part of the tight, highly hydrophobic substrate binding pocket (Fig. 9) that provides the environment needed for catalysis (Schäfer *et al*. 2016).

### How SimC7 binds 7-oxo-SD8

**Figure 10.**
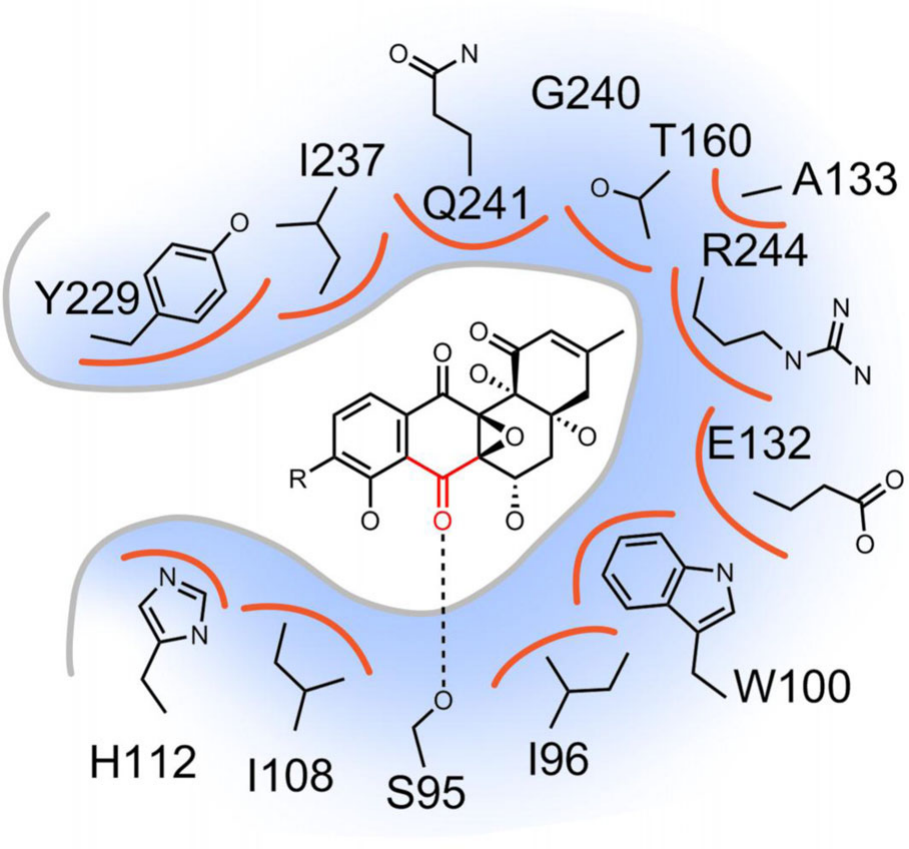
Schematic representation of the hydrophobic substrate-binding pocket of SimC7 showing all residues within 4 Å of the ligand, as revealed in the crystal structure of the SimC7 ternary complex with NADP^+^ and 7-oxo-SD8 (PDB accession number 5L4L). Hydrogen bonds are indicated by dotted lines and van der Waal contacts are indicated by orange arcs. Note that the substrate is bound by only one direct hydrogen bond, connecting the C-7 carbonyl of the angucyclinone and the side-chain hydroxyl of Ser95. This hydrogen bond may assist in positioning the substrate and facilitate the reaction. However, this interaction is not required for enzymatic activity, since a constructed S95A variant of SimC7 shows near wild-type enzymatic activity (Schäfer *et al*. 2016). Note that one face of the pocket is formed by the NADP^+^ cofactor itself. In the natural SimC7 substrate, R = H; in the substrate used here, R includes the deoxysugar, tetraene linker and the aminocoumarin. For clarity, all hydrogens have been omitted.

In the SimC7 ternary complex with substrate and NADP^+^ bound, the angucyclinone ring system of 7-oxo-SD8 binds adjacent and parallel to the nicotinamide ring of the cofactor (Fig. 9c), where it adopts an essentially planar conformation. This differs from the conformations seen in the SimR-SD8 and gyrase-SD8 complexes, where the A-ring of the angucyclinone in SD8 is tilted upwards towards the epoxide (Le *et al*. 2011b, Hearnshaw *et al*. 2014; Schäfer *et al*. 2016). The substrate pocket has several distinctive characteristics (Fig. 9). The pocket is very hydrophobic and highly constricted in shape, features that are likely to enforce the planar conformation on the angucyclinone ring system. Strikingly, within the hydrophobic pocket, 7-oxo-SD8 is bound by just one direct hydrogen bond, connecting the side-chain of Ser95 and the C-7 carbonyl oxygen of the angucyclinone (Fig. 10; Schäfer *et al*. 2016). However, even this single hydrogen bond is not required for enzymatic activity, since a constructed S95A variant shows almost wild-type levels of substrate conversion (Schäfer *et al*. 2016). Thus, although this hydrogen bond may help to position the C-7 carbonyl above the C-4 position of the nicotinamide ring ready for direct hydride transfer, and provide additional polarization of the C-7 carbonyl group, as proposed for the structurally equivalent Ser or Thr residues in classical SDR proteins (Kavanagh *et al*. 2008; Kallberg, Oppermann and Persson 2010; Persson and Kallberg 2013), neither proposed effect is crucial for catalysis. As discussed above, the natural substrate for SimC7 is probably a 7-oxo angucyclinone intermediate lacking the olivose sugar, tetraene linker and aminocoumarin. Consistent with this suggestion, only the angucyclinone is buried in the active site of SimC7, with the rest of the molecule projecting out of the enzyme (Fig. 9). Indeed, the aminocoumarin and roughly half of the tetraene linker are not resolved in electron density.

### How SimC7 converts 7-oxo-SD8 into SD8

**Figure 11.**
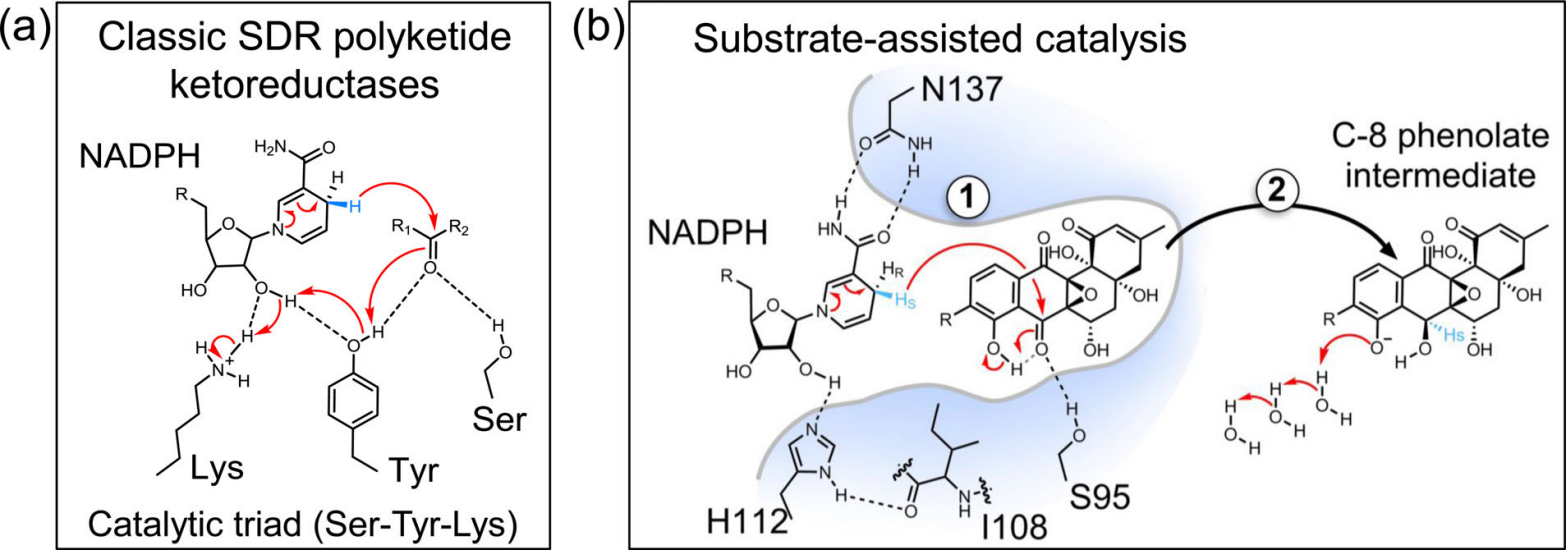
Comparison of the canonical SDR ketoreduction mechanism with the novel SimC7 reaction mechanism. (**a**) In classical SDR proteins, the conserved active site tyrosine serves as a central acid-base catalyst that donates a proton to the substrate. The adjacent lysine residue lowers the pKa of the tyrosine hydroxyl group and often contributes directly to the proton relay mechanism; the hydroxyl group of the serine stabilizes and polarizes the carbonyl group of the substrate. (**b**) SimC7 has an atypical catalytic triad consisting of Ser95, Ile108 and His112. In the first step of the SimC7 mechanism, the C-7 carbonyl group of the angucyclinone is reduced by transfer of the 4-pro-*S* hydride of the cofactor onto the C-7 carbon of the substrate. This transfer from below the C-ring results in the characteristic 7-*S*-stereochemistry of SD8. Ketoreduction at position C-7 is completed by intramolecular proton transfer from the neighboring C-8 hydroxyl group of the angucyclinone; the resultant negative charge on C-8 is stabilized by the adjacent aromatic ring system. In the second step, the C-8 phenolate intermediate regains a proton from bulk water after leaving the substrate binding pocket. In the natural SimC7 substrate, R = H; in the substrate used here, R includes the deoxysugar, tetraene linker and the aminocoumarin. Note that there are no water molecules in the active site pocket that could contribute to the reaction mechanism. In the ternary complex, the nearest water to O-7 of the angucyclinone is ∼5.5 Å away, and the nearest water to O-8 is ∼4.9 Å away. Because of steric constraints within the pocket, neither could approach the substrate oxygen atoms without either a repositioning of the substrate or a conformational change in the protein.

The structures confirmed the prediction made from sequence alignments that SimC7 lacks a canonical SDR Ser-Tyr-Lys catalytic triad (Schäfer *et al*. 2016). While the serine is conserved (Ser95), the other two residues (the YxxxK motif), including the key tyrosine residue that acts as the acid/base catalyst in classical SDR proteins, are replaced by Ile108 and His112, respectively (Fig. 11). The structures also demonstrate that there is no alternative residue that could act as an acid/base catalyst, and instead suggest that SimC7 has a novel reaction mechanism (Schäfer *et al*. 2016). This unusual mechanism does not depend on catalytic residues in the protein, but instead exploits the chemical characteristics of 7-oxo-SD8 itself, and is thus a new example of substrate-assisted catalysis (Dall’Acqua and Carter 2000). In the first step, the hydrophobic environment of the substrate-binding pocket and the juxtaposition of the quinone-like C-ring and the phenyl-like D-ring of the angucyclinone promote the formation of an intramolecular hydrogen bond between the proton on the C-8 hydroxyl and the oxygen of the neighboring C-7 carbonyl (Fig. 11b). This intramolecular hydrogen bond polarizes the carbonyl, enhancing the electrophilicity of C-7 and making it a good acceptor for hydride attack from the 4-*pro-S* position of the nicotinamide ring, which is only 3.0 Å away. Then, internal proton transfer from the neighboring C-8 hydroxyl group forms the C-7 hydroxyl group, generating a phenolate intermediate where the aromatic D-ring stabilizes the negative charge on the C-8 oxygen. In the second step of the reaction, the phenolate intermediate leaves the substrate-binding pocket and the C-8 hydroxyl group re-forms by abstracting a proton from bulk water (Fig. 11b), something that cannot happen within the confines of the active site. The hydrophobic active site cavity would accelerate expulsion of the charged phenolate intermediate created during catalysis. Lastly, the direct hydride attack from below the angucyclinone explains why simocyclinones have 7*S*-stereochemistry. In summary, the SimC7 mechanism involves the intramolecular transfer of a substrate-derived proton to generate a phenolate intermediate, and this obviates the need for proton transfer from a canonical SDR active-site tyrosine.

### Why is 7-oxo-SD8 almost inactive as a DNA gyrase inhibitor?

It is striking that SD8 is very potent as a gyrase inhibitor (IC_50_ ∼ 0.1–0.6 μM) and yet 7-oxo-SD8 is almost inactive (IC_50_ ∼ 50–100 μM) (Schäfer *et al*. 2015). Why does such a small structural difference, the presence of a carbonyl group at the C-7 position in 7-oxo-SD8 (Fig. 8), have such a drastic effect on the antibiotic activity of the molecule? The likely answer becomes clear from analysis of the structure of the GyrA-SD8 complex: both the C-7 and C-8 hydroxyls are involved in a hydrogen bonding network that helps secure the angucyclinone in its binding pocket (Fig. 4). However, in 7-oxo-SD8, an intramolecular hydrogen bond between the C-7 carbonyl and the C-8 hydroxyl is preferred over these intermolecular interactions and this simultaneously breaks the direct contact between the angucyclinone and His80 and the indirect contacts with Pro79 and Arg121 (Fig. 4). His80, in particular, is known to play a crucial role in binding simocyclinone, since mutating this residue to alanine causes a 230-fold increase in the IC_50_ of SD8 for gyrase (Edwards *et al*. 2009b). In addition, the presence of a carbonyl group at C-7 would alter the overall conformation of the angucyclinone ring system, which may well affect other bonding interactions with GyrA.

## CONCLUDING REMARKS

In the three different systems we have described in this review, the interaction of the ligand with the protein has entirely different downstream consequences. For gyrase, it results in inhibition, leading to cell death, for SimR, it results in derepression, leading to antibiotic export, and for SimC7, it results in catalysis, leading to potentiation of an antibiotic. Given that SimC7 is an enzyme, the interaction with the ligand is transient, whereas the interaction with gyrase and SimR will be much longer-lived. In both gyrase and SimR, there are a substantial number of interactions with the terminal aminocoumarin and angucyclinone groups, which are bound by separate subunits; additionally, there are a handful of contacts involving the linker region. The extensive nature of these double-headed interactions leads to very tight binding, commensurate with the physiological consequences. Indeed, molecules lacking either the angucyclinone or the aminocoumarin bind much more weakly to DNA gyrase and, as a consequence, the potency of SD8 as an antibiotic is severely compromised through loss of either ‘warhead’ (Edwards *et al*. 2009b). The proportion of hydrogen bonds is highest for the complex with gyrase because the binding site is largely solvent-exposed and would otherwise interact with the G-segment DNA, which is polar. In SimR, the ligand-binding site threads through the hydrophobic core of the homodimer, and so the interactions are dominated by van der Waals contacts. In contrast, in the 7-oxo-SD8 complex with SimC7, only the angucyclinone interacts with the enzyme, this being consistent with the site of ketoreduction and the expectation that the natural substrate *in vivo* is the angucyclinone alone. Given the necessity to precisely position the SimC7 substrate for catalysis, the dearth of hydrogen bonds seems counterintuitive. Indeed, a Ser95 to Ala substitution that removes the only hydrogen bond shows that even this is dispensable. However, the necessity to provide a hydrophobic environment for efficient catalysis would be consistent with a paucity of hydrogen bonding partners and bound water molecules. Instead, the highly constrained nature of the SimC7 active site is a key factor in sterically guiding the substrate to its catalytically competent position adjacent to the cofactor with hydride donor and hydride acceptor atoms juxtaposed. The transient nature of this interaction would be promoted by the negative charge that develops on the phenolate intermediate, which would be unfavorable in the hydrophobic active site, and possibly also by the increased puckering of the angucyclinone ring system that would occur when the C7 keto group is reduced to a hydroxyl.

Finally, although SD8 itself is not viable as a clinical antibiotic, due at least in part to its poor penetration into bacteria, the way in which it inhibits DNA gyrase is unique. It therefore has the potential to guide the development of new, clinically relevant compounds acting against this enzyme, and the detailed structural information available should potentiate such development.
